# Wheat Spike Detection and Counting in the Field Based on SpikeRetinaNet

**DOI:** 10.3389/fpls.2022.821717

**Published:** 2022-03-03

**Authors:** Changji Wen, Jianshuang Wu, Hongrui Chen, Hengqiang Su, Xiao Chen, Zhuoshi Li, Ce Yang

**Affiliations:** ^1^College of Information and Technology, Jilin Agricultural University, Changchun, China; ^2^Institute for the Smart Agriculture, Jilin Agricultural University, Changchun, China; ^3^College of Food, Agricultural and Natural Resource Sciences, University of Minnesota, Paul, MN, United States; ^4^Key Laboratory of Bionic Engineering, Ministry of Education, Jilin University, Changchun, China

**Keywords:** wheat spikes, detection and counting, deep learning, attentional mechanism, wheat yield

## Abstract

The number of wheat spikes per unit area is one of the most important agronomic traits associated with wheat yield. However, quick and accurate detection for the counting of wheat spikes faces persistent challenges due to the complexity of wheat field conditions. This work has trained a RetinaNet (SpikeRetinaNet) based on several optimizations to detect and count wheat spikes efficiently. This RetinaNet consists of several improvements. First, a weighted bidirectional feature pyramid network (BiFPN) was introduced into the feature pyramid network (FPN) of RetinaNet, which could fuse multiscale features to recognize wheat spikes in different varieties and complicated environments. Then, to detect objects more efficiently, focal loss and attention modules were added. Finally, soft non-maximum suppression (Soft-NMS) was used to solve the occlusion problem. Based on these improvements, the new network detector was created and tested on the Global Wheat Head Detection (GWHD) dataset supplemented with wheat-wheatgrass spike detection (WSD) images. The WSD images were supplemented with new varieties of wheat, which makes the mixed dataset richer in species. The method of this study achieved 0.9262 for mAP50, which improved by 5.59, 49.06, 2.79, 1.35, and 7.26% compared to the state-of-the-art RetinaNet, single-shot multiBox detector (SSD), You Only Look Once version3 (Yolov3), You Only Look Once version4 (Yolov4), and faster region-based convolutional neural network (Faster-RCNN), respectively. In addition, the counting accuracy reached 0.9288, which was improved from other methods as well. Our implementation code and partial validation data are available at https://github.com/wujians122/The-Wheat-Spikes-Detecting-and-Counting.

## Introduction

As one of the three major cereal crops, wheat provides food for approximately one-third of the world’s population. Global wheat consumption has increased due to rising per capita income and urbanization. On the other hand, wheat crops are increasingly being hampered by phenological changes, shrinking germplasm areas, and other stresses. Therefore, wheat genetic improvement is critical to address future food security. At present, most wheat cultivation and breeding researchers rely on costly manual counting. This time-consuming process is driving the need for new tools. In addition, subjectivity and fatigue will lead to mistakes in counting wheat spikes (Jin et al., 2017). When assessing crop genetic improvement, although genotyping is easier and more accurate than before, efficient phenotyping algorithms and techniques still limit the establishment of phenotype-genotype relationships (Eversole et al., 2014). Therefore, the construction of efficient phenotypic algorithms and technologies are particularly urgent and necessary for improving genetic efficiency. Furthermore, wheat yield is one of the important indexes of quality breeding. So, the detection and counting of spikes efficiently are one of the main research directions of phenotypic technology based on phenotype-genotype relationships for crop production (Slafer et al., 2014; Ferrante et al., 2017).

In the past decade, image processing has been increasingly used in analyzing and extracting phenotypic parameters. Features that include color, texture, shape, and edge are fused in the classifier to detect wheat spikes using traditional image processing methods. Mirnezami et al. (2020) compared automated and semiautomated soybean trichome counting methods, which used thresholding and graph algorithms based on color and shape features. They achieved approximately 90% accuracy using semiautomated annotation, which outperformed manual counting. Kulkarni and Patil (2012) employed the Gabor filter to detect plant diseases by extracting typical plant features from red–green–blue (RGB) images, including texture, edge, and color for plant disease segmentation. Then, the features were used to train the artificial neural network, and the accuracy reached 91%. Sun et al. (2019) applied a region growing algorithm with a double threshold integrating spatial and color features to segment cotton bolls and developed an algorithm based on geometric features to count cotton bolls. The counting accuracy was 84.6%, and the F1 score was approximately 98%. The panicle segmentation method extracted the color and texture of the panicles to realize (semi) automatic counting of wheat spikes (Cointault et al., 2008). Fernandez-Gallego et al. (2018) presented an automatic spike-counting method to calculate the number of spikes based on color images taken under natural conditions. Additionally, the local peaks are segmented and counted by the color features and the Find Maxima. The results showed that the accuracy of wheat spikes counting is 90%, and the standard deviation is 5%. Although most of these studies achieved good results, there were still problems. They have used the traditional image processing method and therefore require manual screening of features. This limitation hinders the popularization and application of the algorithm in more complex problems. The wheat spike detection and counting is still a very challenging task.

Deep learning performs exceptionally well in detection and classification tasks. A series of novel deep learning models have been developed, such as region-based convolutional neural network (R-CNN), Fast R-CNN, Faster R-CNN, fully convolutional one-stage object detector (Fcos), You Only Look Once (Redmon et al., 2016; Redmon and Farhadi, 2017) version 3 (Yolov3), You Only Look Once version4 (Yolov4), You Only Look Once version 5 (Yolov5), RetinaNet, and single-shot multiBox detector (SSD) (Girshick et al., 2014; Girshick, 2015; Ren et al., 2015; Liu et al., 2016; Lin et al., 2017a; Redmon and Farhadi, 2018; Tian et al., 2019, Bochkovskiy et al., 2020; Jocher et al., 2020), which are ready to be used in phenotyping applications. Backbone network and feature pyramid network (FPN) (Lin et al., 2017b) are the two main components of an object detection framework. The backbone network conducts feature extraction, whereas FPN conducts feature fusion. As a result, advancements in the backbone network and FPN directly impact the performance of the object detection network. He et al. (2016) proposed residual network (ResNet), introducing residual blocks and realizing across layer information transmission through shortcut connections resulting in improved optimization. After that, many studies designed various modules to strengthen the ability of network feature extraction. For example, selective kernel (SK) block (Li et al., 2019), squeeze-and-excitation (SE) block (Hu et al., 2018), non-local block (Wang et al., 2018), convolutional block attention module (CBAM) (Woo et al., 2018), split attention block (Zhang et al., 2020), etc. FPN fuses multiscale features extracted through deep convolutional networks. Tan et al. (2020) proposed a simple and efficient feature pyramid structure to address the top-down architecture of FPN, which is called a bidirectional feature pyramid network (BiFPN). It allows top-down and bottom-up multi-scale weighted feature fusion.

Wheat spike image sets, such as ACID (Pound et al., 2017) and SPIKE (Hasan et al., 2018) were used in many studies and they achieved good deep learning model training results (Alkhudaydi and Zhou, 2019; Madec et al., 2019; Yang et al., 2019). Misra et al. (2021) developed an online platform “Web-spikeSegNet” that uses deep learning methods to segment wheat spike images taken under laboratory environment conditions. It can achieve 99.59% segmentation accuracy. Zhao et al. (2021) proposed an improved Yolov5 network by adding a microscale detection layer, setting prior anchor boxes, and adapting the confidence loss. These improvement points solve spike error detection and miss detection caused by occlusion conditions in UAV images. These studies used deep learning methods to overcome the disadvantages of traditional image processing methods that require manual feature design. However, the datasets used in these studies are relatively homogeneous in terms of wheat spike collection environments and varieties. Most wheat spike datasets are limited in terms of genotype, geographic area, and observational condition. Therefore, the research requires a richer dataset and the ability to overcome the detection of wheat spikes in complex environments. The Global Wheat Detection (GWHD) dataset (David et al., 2020) was a standard image set collected by several research institutions, which was considered by many scholars as a new challenge for wheat spike detection. Bhagat et al. (2021) proposed a novel WheatNet-Lite network, which was solved the dense and overlapping wheat spikes. The network was validated on GWHD, SPIKE, and ACID datasets. The mAP50 values were 91.32, 86.10, and 76.32%, respectively. Li et al. (2021) also investigated the GWHD dataset. They trained RetinaNet models using migration learning. The images of wheat at the filling stage and the maturity stage from the GWHD dataset were used for regression analysis of count results. The *R*^2^ was 0.9722. Wang et al. (2021) proposed an occlusion robust wheat spike counting algorithm based on EfficientDet-D0 with the CBAM attention module. It was the network that focused more on small wheat spikes with the counting accuracy which was 94% and the false detection rate was 5.8% on the GWHD dataset. The new models in these studies were proposed to solve the wheat spike images occlusion problem. However, it is not only the occlusion images of wheat spikes that are difficult to recognize in the field, but also difficult to recognize wheat spike images with dim lighting and complex environmental backgrounds. Therefore, there is still room for continued improvement in wheat spike detection and counting. In this study, we used the GWHD dataset supplemented with wheat-wheatgrass spike detection (WSD) images, where WSD was collected from trials. There is one variety in our dataset, Jilin wheat-wheatgrass No. 37. Because of its excellent quality, wheat-wheatgrass has been crowned as a geographical landmark product of Jilin Province. The spike of wheat-wheatgrass No. 37 is rectangular in shape, and the spike length is usually 10–12 cm. The wheat spikes have white hulls and are awned but without hairs. WSD images added diversity to the GWHD to train spike detection models.

In this study, SpikeRetinaNet was trained to detect wheat spikes based on the RetinaNet network structure of a one-stage detector, which kept the one-stage detector’s speed while improving detection accuracy. In the dataset, it is difficult to distinguish wheat spikes because of light, shadows, color, and shape similarity. To solve the problems, the focal loss function was introduced into the structure of RetinaNet to reduce the influence of background during wheat spike detection tasks (Lin et al., 2017a). Meanwhile, we introduced the BiFPN (Tan et al., 2020) and double SA (DSA) (split attention block and spatial attention block) into the backbone network to realize fine-grained feature extraction and representation across feature map groups and strengthen the fusion of global information and local information. By proposing BiFPN, it introduces learnable weights to learn the importance of different input features and repeatedly applies top-down and bottom-up multiscale feature fusion. Because different input features have different resolutions, their contribution to the fused fine-grained features is different. Meanwhile, introducing DSA into the backbone realizes the interaction between feature map channels and receptive field regions. In this way, fine-grained discriminant feature of detecting wheat spikes, such as the shape, texture, and color, can be better extracted and represented. The cluster growth of wheat spikes makes it difficult to distinguish between multiple wheat spikes or multinode parts of wheat spikes because wheat spikes occlude each other. In the previous work, non-maximum suppression is an integral part of the object detection pipeline which is used to filter the detection candidate boxes. The detection box with the maximum score is selected and all other detection boxes with a significant overlap (using a predefined threshold) are suppressed. To this end, we introduced soft non-maximum suppression (Neubeck and Van Gool, 2006) (Soft-NMS) (Bodla et al., 2017), an algorithm that decays the detection scores of all other objects as a continuous function of their overlap, to solve the problem of missed detection caused by mutual occlusion.

## Materials and Methods

### Image Data Acquisition

**FIGURE 1:**
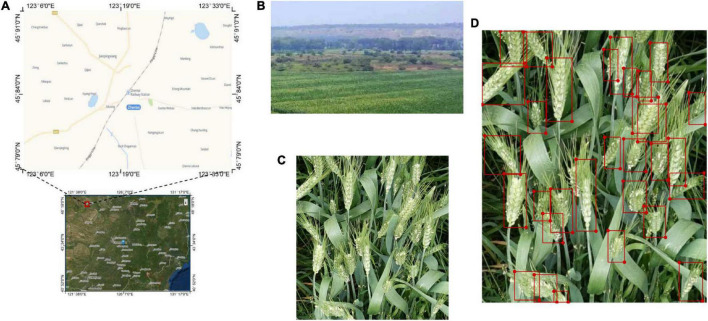
Data acquisition and labeling process: **(A)** map of data collection place; **(B)** the WSD images collection scene; **(C)** an example WSD image; **(D)** annotation of a WSD images.

The original GWHD dataset included 4,700 high-definition color images of wheat from multiple genotypes. There were a total of 190,000 wheat spikes annotated. Wheat spikes in the image were labeled interactively by delimiting bounding boxes that contained all spike’s pixels using web-based labeling (Brooks, 2019). Seven categories that contain 3,373 images and 147,793 labeled spikes from Europe and North America were used in this article. The seven categories are Arvalis_1, Aralis_2, Aralis_3, INRAE_1, USask_1, RRes_1, and ETHZ_1. They are collected between 2016 and 2019. They were acquired over experiments following different growing practices, with row spacing varying from 12.5 cm (ETHZ_1) to 30.5 cm (USask_1). They include normal sowing density (Arvalis_1, Arvalis_2, Arvalis_3, and INRAE_1) and high sowing density (RRes_1 and ETHZ_1). The GWHD dataset covers a range of pedoclimatic conditions including very productive contexts, such as the loamy soil of the Picardy area in France (Arvalis_3), silt-clay soil in mountainous conditions, such as the Swiss Plateau (ETHZ_1) or Alpes de Haute Provence (Arvalis_1 and Arvalis_2). In the case of Arvalis_1 and Arvalis_2, the experiments were designed to compare irrigated and water-stressed environments. An average of 44 spikes was present in each image, with a range of 15–70 real spikes per image. The WSD images that contain 210 high-definition color images and 6,123 annotations were used in this study to supplement the experimental data as well. All images were collected from Chengkai Cooperative, Nangangzi Village, Zhenlai Town, Baicheng City, Jilin Province, China (45.83 N, 123.21 E) from May to July 2020 using a Canon 11 EOS 80D digital camera. Images were captured at the height of 30–70 cm above the wheat canopy and at various tilt angles. The resolution of the WSD images was 3,456 × 4,408 pixels. All images were stored in JPG format according to the RGB color standard. Then, the collected images were labeled by the LabelImg Tool (LabelImg, 2015). The overall process is shown in Figure 1.

### Formation of the Mixed Dataset

**FIGURE 2:**
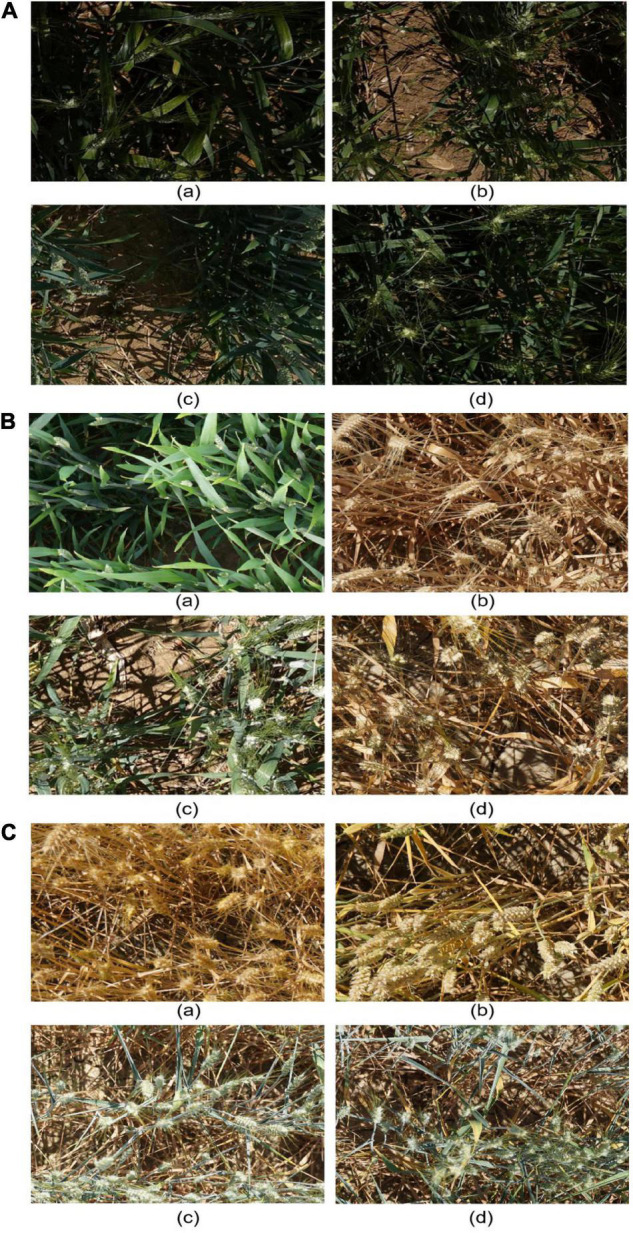
Sample images taken under different complex conditions. **(A)** Dim illumination conditions. **(B)** The complex environment illumination conditions. **(C)** Overlapping occlusion of wheat spikes.

The original image set was first normalized to obtain a total of 3,583 images of 1,024 × 1,024 pixels due to the limited computing power of laboratory equipment. The diversity and complexity of the mixed dataset brought great difficulties to the method in detecting and counting. Three image categories were the most difficult to identify: (1) images with low illumination, (2) complex environment, and (3) overlapping objects. For example, it is difficult to distinguish wheat spikes in the evening due to dim light and complicated shadows (Figure 2A). When wheat plants are young (Figure 2B(a)), their spikes are small and as green as the leaves. Wheat spikes (Figure 2B(b,d)) and stems are similar in color too, and there is a mutual occlusion phenomenon, which can easily confuse analysis. Figure 2B(c,d) is sparsely planted, with a visible soil background, and the distribution of shadows is mixed by light. The cluster growth of wheat spikes in Figures 2C(a,b) makes it difficult to distinguish between multiple wheat spikes or multinode parts of wheat spikes. In Figures 2C(c,d), wheat spikes occlude each other, which make it difficult to mark.

Our next step filtered out some inappropriate bounding boxes [the boundary box is too large (box areas >200,000) or too small (box areas <50)] from the dataset before putting the images into the model to make it more accurate and clean. Then, we used online augmentation techniques, such as horizontal and vertical flips, rotations and resizing, and augmenter and normalizer to enhance the image. This method has the advantage of not requiring the augmented data to be synthesized, which saves data storage space and provides high flexibility. Among the 3,583 wheat images collected, 70% of each category in the mixed dataset was extracted as the training dataset, 20% of images were extracted as the validation set, and 10% of images were extracted as the test set.

### Overall Design of the SpikeRetinaNet

**FIGURE 3:**
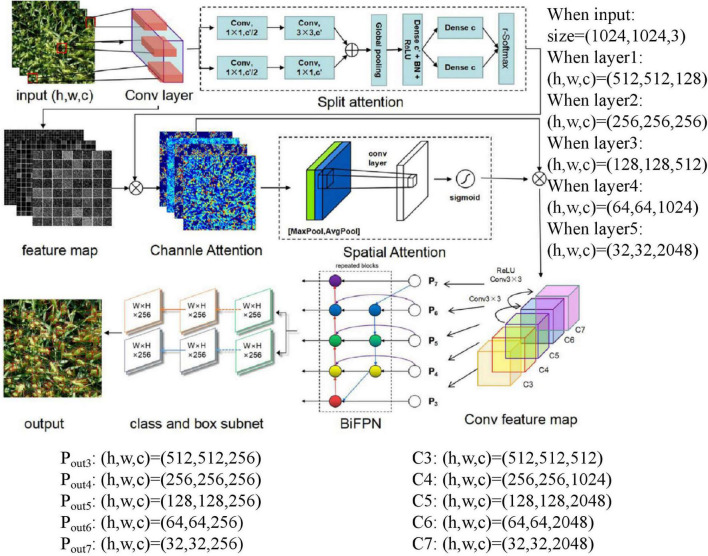
The schematic layout of the SpikeRetinaNet for the robust detection and counting of wheat spikes.

Figure 3 depicts the specific process of our proposed SpikeRetinaNet. First, the image features are extracted through the convolution layer. Then, the extracted feature sets are grouped and convoluted to calculate the weight of the feature channel and then performing a weighting operation on the obtained weights and feature sets. Second, we perform AdaptiveAvgPool2d and AdaptiveMaxPool2d on the results obtained. We then use the sum of the pooling results to calculate the weight value through the Sigmoid function and then performing another weighting operation on the weight value and feature set to get the result of spatial attention. Third, SpikeRetinaNet employs five levels of feature pyramids. P3, P4, and P5 are calculated by top-down and lateral connections of the corresponding backbone network’s C3, C4, and C5 layers (architectures for ImageNet (Krizhevsky et al., 2012) are divided into C1–C5), respectively. P6 is obtained by upsampling based on C5, and ReLU obtains P7 based on P6. The output is obtained by weighted bidirectional calculation of P3–P7. Finally, the results of each layer of the FPN are input into two subnetworks of classification and regression, respectively, to get the final output image.

#### RetinaNet

RetinaNet is an object detector that consists of a backbone network and two task-specific subnetworks. Among them, the backbone networks include a convolutional neural network to extract information from the image and the FPN enhancing the feature information with top-down and lateral connections. The two subnets use convolution to classify and regress from bounding boxes to real object boxes.

The core of RetinaNet is focal loss. It simply and efficiently solves the category imbalance faced by the one-stage detector, which improves the classification precision of the one-stage detector. RetinaNet was proposed to reshape the standard crossentropy loss to focal loss to deal with the category imbalance. It downweighs simple samples so that even if the number of samples is large, their contribution to the total loss is small. The focal loss formula is as follows Eq. 1.


(1) $$\begin{array}{c} F\,L\,\left(p_{t}\right)=-\alpha \,t\,{\left(1-p\,t\right)}^{\gamma }\,\log\left(p\,t\right)\\ p\,t=\left\{ \begin{array}{c} p\;i\,f\,\gamma =1\\ 1-p\;o\,t\,h\,e\,r\,w\,i\,s\,e \end{array}\right. \end{array}$$

The weighting factor α ∈ [0,1] is the parameter for class *1*, and 1−α for class –1, α maybe set by inverse class frequency or treated as a hyperparameter set by cross-validation. Though α balances the weight values of positive or negative examples, it does not differentiate between easy or hard examples. So, the modulating factor (1−*pt*)^γ^ is introduced with a tunable focusing parameter and γ≥0 and *pt* is the class probability score. The proposed adjustment factor reduces the loss weights ratio from simple examples and quickly focuses on hard examples. It is suitable for difficult distinguishing between foreground and background, such as many negative examples in the process of wheat spike detection. Therefore, when discussing dense object detection (such as our mixed dataset), RetinaNet is the best choice for speed and accuracy.

#### Selection of the Feature Learning Network

**FIGURE 4:**
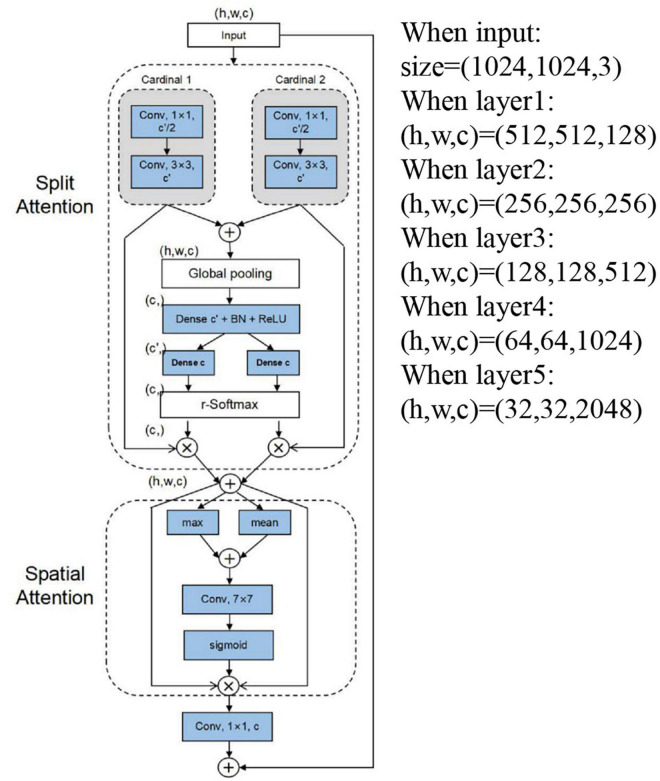
Schematic layout outline of ResNet-DSA.

The design of the feature learning network is very important. We add the DSA (double SA, split attention block, and spatial attention block) to the backbone network of RetinaNet to enable the feature mapping attention between different feature mapping groups and emphasize the spatial location information. Further detailed description in Figure 4 divides the features into two groups (*V*_*1*_ and *V*_*2*_) for 1 × 1 convolution followed by a 3 × 3 convolution. The attention weight is parameterized using two fully connected layers with ReLU activation. We aggregate channel information of a feature map using two pooling operations (maxpool and avgpool), generating two 2D maps. Then, we connect them and convolute them through standard convolution operation to form our 2D spatial attention maps. Finally, if the input and output feature maps have the same size, the final output *Y* of our DSA is produced using a shortcut connection: *Y* = *V* + *X* (*V* = *Cancat*{*V*_1_,*V*_2_}). For blocks with a stride, an appropriate transformation *T*(*X*) is applied to the shortcut connection to align the output shapes: *Y* = *V* + *T*(*X*). The specific shape is depicted in the note of Figure 4, where the feature maps become smaller and the channels become more numerous as the network depth deepens. The backbone network has better and more accurate feature extraction capabilities than ResNet. Therefore, we can extract more detailed features for the spike of wheat detection. For the problem of dim light and complex environment background in the mixed datasets, we can apply the DSA attention module to emphasize the characteristics of wheat spikes. Similarly, suppose the wheat spikes in the data set are similar to the background. In that case, we can also use the attention block to emphasize the useful features and suppress the useless features.

#### Design of the Feature Pyramid Network Backbone

We use BiFPN of FPN to enhance feature fusion. BiFPN can realize fast bidirectional cross-scale connections and weighted feature fusion. Among them, multiscale feature fusion is to be carried out using different levels and different resolutions of the input. This produces a list of multiscale features Pi⁢n→i⁢n=(Pl1i⁢n,Pl2i⁢n,…), which $P_{l_{i}}^{i\,n}$ represents the feature at a level *l*_*i*_. BiFPN requires $P_{3}^{i\,n}$ to $P_{7}^{i\,n}$ level inputs for aggregate features. The traditional output calculation of FPN is shown in Eq. 2, where *Resize* is the upsampling or downsampling operators to adjust the image size and *Conv* is a convolutional operator.


(2) $$\begin{array}{c} P_{7}^{o\,u\,t}=C\,o\,n\,v\,\left(P_{7}^{i\,n}\right)\\ P_{6}^{o\,u\,t}=C\,o\,n\,v\,\left(P_{6}^{i\,n}+\mathrm{Resize}\,\left(P_{7}^{\text{out}}\right)\right)\\ \cdots \\ P_{3}^{o\,u\,t}=C\,o\,n\,v\,\left(P_{3}^{i\,n}+\mathrm{Resize}\,\left(P_{4}^{\text{out}}\right)\right) \end{array}$$

Therefore, BiFPN adds an extra bottom-up path aggregation network to solve the problem that conventional FPN only has top-down unidirectional information flows. Besides, the bidirectional network is simplified by removing the node with only one input channel to integrate more features without increasing much cost. Therefore, we represent the fused feature at level six for BiFPN shown in Eq. 3:


(3) $$\begin{array}{c} P_{6}^{t\,d}=C\,o\,n\,v\,\left(\frac{\omega_{1}\cdot P_{6}^{i\,n}+\omega_{2}\cdot \text{Resize}\,\left(P_{7}^{i\,n}\right)}{\omega_{1}+\omega_{2}+\varepsilon }\right)\\ P_{6}^{\text{out}}=C\,o\,n\,v\,\left(\frac{\omega_{1}^{\prime }\cdot P_{6}^{i\,n}+\omega_{2}^{\prime }\cdot P_{6}^{t\,d}+\omega_{2}^{\prime }\cdot \text{Resize}\,\left(P_{5}^{\text{out}}\right)}{\omega_{1}^{\prime }+\omega_{2}^{\prime }+\omega_{3}^{\prime }+\varepsilon }\right) \end{array}$$

$P_{6}^{t\,d}$ is the median feature at level six on the top-down pathway and $P_{6}^{\text{out}}$ is the output feature at level six on the bottom-up pathway. The bidirectional fusion of BiFPN deepens the degree of feature fusion. So, in the mixed dataset, images with complex environment backgrounds can use deep, low-resolution, and high semantic features to distinguish wheat spikes and background. As a result, more overlapping wheat spikes can be retained. Meanwhile, shallow, high-resolution features could provide more accurate location information. It can also locate the problem of wheat spikes occlusion better.

#### Soft Non-maximum Suppression

Soft non-maximum suppression was introduced to obtain consistent improvements for the selection of candidate boxes. Soft-NMS suppresses overlapping boxes with a non-maximum value and sets the attenuation function for near boxes based on the overlapping boxes’ size instead of setting its score to zero. Intuitively, if the crossarea between the bounding box and *M* is higher than the threshold, its score should be reduced. If its overlap is lower than the threshold, it keeps the detection score unchanged. The calculation formula is shown in Eq. 4, where *S*_*i*_ is the final score, *i* is the subscript, *M* is the box with the highest score in the prediction box set, *b*_*i*_ is the box in the prediction box set *B*, and *N*_*t*_ is the intersection-over-union (IoU) threshold of *M* and *b*_*i*_. The formula Eq. 5 updated the pruning step with the following rule. Under natural conditions, the presence of wheat spikes occlusion is inevitable in wheat spikes data collection. The Soft-NMS can effectively retain the blocked wheat spikes without affecting the selection of the normal calibration box.


(4) $$S_{i}\,\left\{ \begin{array}{c} S_{i},i\,o\,u\,\left(M,b_{i}\right)<N_{t}\;\\ S_{i}\,\left(1-i\,o\,u\,\left(M,b_{i}\right)\right),i\,o\,u\,\left(M,b_{i}\right)\geq N_{t}\; \end{array}\right.$$

(5) $$S_{i}=S_{i}\,e^{-\frac{i\,o\,u\,{\left(M,b_{i}\right)}^{2}}{\sigma }},\forall b_{i}\notin D$$

See Table 1 for detailed architectures compared the SpikeRetinaNet with the original RetinaNet.

**TABLE 1:** Architectures for ImageNet.

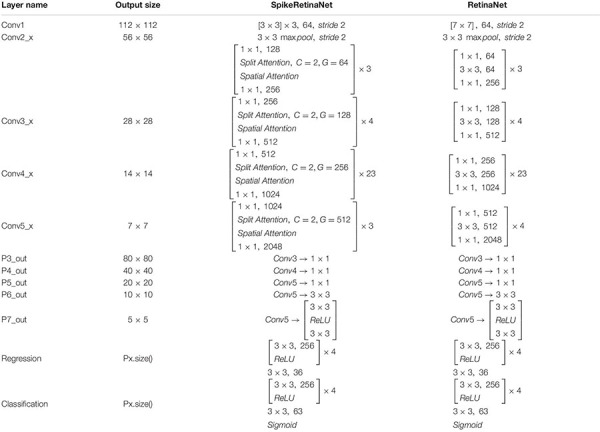

*Building blocks are shown in brackets, with the numbers of blocks stacked. C is the number of groups, and G is the number of channels per group.*

## Training the Wheat Spike Detecting and Counting Model

### Computational Hardware and Platform

All processing experiments in this article were carried out by the DELL Precision T7920 Tower deep learning workstation which consisted of an Intel(R) Xeon(R) Gold 5218 CPU with a clock speed of 2.1 GHZ, 62.5 GB DRAM, 503 GB hard disk, and a GeForce RTX 2080 Ti/PCIe/SSE2 graphics card. The operating environment was Ubuntu 18.0.4, Pytorch = 1.7.0, Python 3.7.

### Model Training

The SpikeRetinaNet was used in the mixed dataset training process. First, DSA was used to extract the features from the backbone network. Second, BiFPN improved the extracted feature map by adding more expression and multiscale target region data. Finally, two subnetworks with the same structure but no shared parameters used BiFPN feature maps to complete the object classification task and regress the offset from the bounding box to a nearby real object. The Soft-NMS was also used to choose calibration boxes. The parameters of the different layers are described in the note of Figure 3. The specific algorithm flow is as follows:

Input: image to be detected D.

Output: Vector C is used for sample categories, and *R* is used for boundary coordinates.

**Step 1:** Convolution layer for feature extraction. First, 64 convolution kernels with 7 × 7 stride-2 are used to feature extraction, and then, a maxpooling with 3 × 3 stride-2 is used to get the feature set. Second, all feature maps are divided into 2 splits. Additionally, the split attention is used to calculate the weight of each split, and the weighted feature maps are used as the input of the spatial attention module. Finally, a 1 × 1 Conv is used again to change the number of channels and use skip connection to fuse the original input features of a DSA block (the fusion method is element-wise sum). There are 101 layers as a feature extraction network.

**Step 2:** FPN, the multiscale features formed in the backbone network, is input into the feature pyramid for enhancement and utilization, and the feature map with stronger expression and multiscale target information is obtained. The backbone network is divided into C1–C5 layers. Add a 1 × 1 Conv on C5, and the upsampling is two times as much to generate the feature map, and then, a ReLU activation function is performed to form $P{}_{7}^{\text{td}}$. $P{}_{6}^{\text{td}}$ is to add a 1 × 1 Conv on C5, and the upsampling is two times as much to generate the feature map and then fuse with $P{}_{7}^{\text{td}}$. $P{}_{5}^{\text{td}}$ is directly mapped from C5 to merge $P{}_{6}^{\text{td}}$ upsampling. $P{}_{4}^{\text{td}}$ and $P{}_{3}^{\text{td}}$ have the same structure as $P{}_{5}^{\text{td}}$. $P{}_{3}^{\text{td}}$ to $P{}_{7}^{\text{td}}$ is the input of the FPN. $P{}_{3}^{\text{out}}$ is upsampled by C3 fusion $P{}_{4}^{\text{td}}$, $P{}_{4}^{\text{out}}$ is formed by $P{}_{4}^{\text{td}}$ and C4 fusion $P{}_{3}^{\text{td}}$ downsampling. $P{}_{5}^{\text{out}}$ and $P{}_{6}^{\text{out}}$ have the same structure as $P{}_{4}^{\text{out}}$. $P{}_{7}^{\text{out}}$ is downsampled and fused by $P{}_{6}^{\text{td}}$ and $P{}_{6}^{\text{out}}$. Finally, a 3 × 3 Conv stride-2 is used for all the layers obtained after fusion to eliminate the aliasing effect of upsampling.

**Step 3:** The output of each layer of the feature pyramid performs two subnetwork tasks (classification and boxes regression). Each subnetwork uses four layers of 3 × 3 × 256 Conv and then connects to 3 × 3 × KA (K is the number of object classes, A = 9 anchors per level) Conv. In addition, it finally uses Sigmoid activation to the output KA binary predictions at each spatial position.

**Step 4:** Use a trained model to perform the next decoding process on the top 1,000 boxes with the highest scores on each FPN level. Summarize boxes of all levels, filter boxes with a soft threshold of 0.1, and finally get the final boxes location of the target. The training loss is composed of boxes position information L1 loss and category information Focal-Loss. Considering the extreme imbalance between positive and negative samples when the model is initialized, the bias parameter of the last convolution is initialized.

The specific steps of the training are as follows: due to equipment limitations, a minibatch of four images will be used to train the model. The Optimizer selects Adam, uses Reduce LROnPlateau to dynamically adjust the learning rate, the initial learning rate is 1e-4, and uses all images of the training dataset to train 100 epochs to analyze the training process. Additionally, the same platforms are also applied to faster region-based convolutional neural network (Faster-RCNN), YoLov3, YoLov4, YoLov5s, YoLov5m, and SSD, which codes are publicly available for comparison.

### Network Evaluations

For this study, all samples were divided into four types according to the IoU between the predicted bounding boxes and the real bounding boxes exceeding a given parameter. True positive (TP) corresponds to the correct predicted bounding boxes. False-positive (FP) corresponds to the erroneously predicted bounding boxes. False-negative (FN) is the marked bounding box that could not be detected. Otherwise, it is a true negative (TN). Eq. 6 precision (P) and Eq. 7 recall (R) are computed.


(6) $$P=\frac{T\,P}{T\,P+F\,P}$$

(7) $$R=\frac{T\,P}{T\,P+F\,N}$$

Since the evaluation index mainly focuses on the positive sample, thus to weigh the precision index and the recall index, *AP*_k_ (the value *k* represents the type of wheat spikes) was defined in Eq. 8 as the area under the *P*_*k*_ and *R*_*k*_ curve of the class *k*. *AP* is a standard measure to measure the sensitivity of the network to target objects, and it is also an indicator of the overall performance of the network. Additionally, m*AP* was defined in Eq. 9 as the average precision of the eight classes of wheat spikes. The higher the m*AP*, the better the detection results of the convolutional neural network for the object detection, and the average detection time is also calculated to evaluate the performance of the model.


(8) $$A\,P_{k}=\int_{0}^{1}P\,\left(R_{k}\right)\,dR_{k}$$

(9) $$m\,A\,P=\frac{1}{8}\,\sum_{k=1}^{8}A\,P_{k}$$

Two other metrics were proposed to evaluate the performance of spikes counting: root mean square error (RMSE) as Eq. 10 and root mean square percentage error (RMSPE) as Eq. 11, which *N*_*p*_ is the predicted value of wheat spikes and *N*_*g*_ is the actual value of wheat spikes. The number of spikes detected by the model and the number of spikes counted manually were analyzed by simple linear regression. The coefficient of determination *R*^2^ was calculated to assess the effectiveness of using one variable to predict the other.


(10) $$R\,M\,S\,E=\sqrt{\frac{1}{k}\,\sum_{i=1}^{k}{\left(N_{p}-N_{g}\right)}^{2}}$$

(11) $$R\,M\,S\,P\,E=\sqrt{\frac{1}{k}\,\sum_{i=1}^{k}{\left|\frac{\left(N_{p}-N_{g}\right)}{N_{p}}\right|}^{2}}$$

## Results

### Ablation Study

#### Evaluation of the SpikeRetinaNet

**FIGURE 5:**
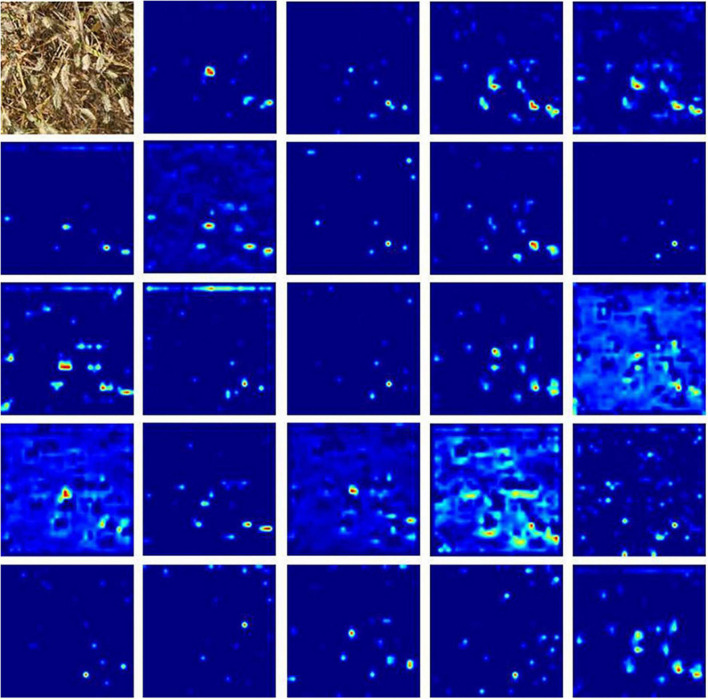
The first image is the original image, the other images are the class activation mapping (CAM) for the different feature channels of our method.

**FIGURE 6:**
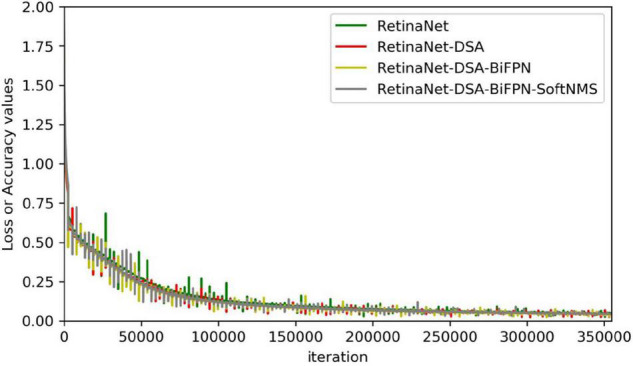
Ablation study loss.

In this subsection, we empirically show the effectiveness of our design choice. As shown in Table 2, the results indicate that the effect of added DSA blocks to RetinaNet is better than the original network, and the mAP is increased by 4.40%. The RetinaNet-BiFPN is better than RetinaNet-FPN, and the mAP is increased by 1%. Therefore, our model can improve the mAP of RetinaNet by about 5.40%. The class activation mapping (Selvaraju et al., 2017) of our model is shown in Figure 5. The CAM uses the gradient information from the feature map from the P7 layer of the BiFPN to understand the importance of each feature point to the target decision. The thermodynamic features of different colors reveal the “attractiveness” of the regional network. Among them, the red area represents the most significant influence on the network. As the color changes from red to yellow and finally to blue, it means that the influence has decreased. So, in Figure 5, these 24 images represent the visualization result of the 24 feature channels (partial feature channel of P7 layer of BiFPN), thus reflecting our method can focus on wheat spike features in the complex environment. The results show that our backbone has a better capability of feature extraction. Finally, we improve the NMS parts, using Soft-NMS to select candidate boxes, and the performance is improved by 5.59%. The network complexity of our method is increased, so the FPS is reduced from 35 to 22, the increase of time is not much, and the performance is improved significantly. As shown in Figure 6, the convergence rate of the loss value is similar in the self-verification comparison experiment, but the fluctuation of RetinaNet is the largest, and our method is the most stable.

**TABLE 2:** Mean average precision (mAP), frames per second (FPS), root mean square error (RMSE), and root mean square percentage error (RMSPE) of RetinaNet in detecting wheat spikes.

Method	Datasets	mAP50	mAP75	FPS	RMSE	RMSPE	Counting Acc
RetinaNet	Mixed	0.8703	0.4701	35	2.63	0.08	0.8984
RetinaNet-DSA	Mixed	0.9143	0.4842	30	–	–	0.9122
RetinaNet-DSA-BiFPN	Mixed	0.9243	0.4942	25	–	–	0.9206
**Our method**	**Mixed**	**0.9262**	**0.5023**	**22**	**1.96**	**0.06**	**0.9228**

*The results of our method are highlighted in bold.*

#### Counting Strategy

**FIGURE 7:**
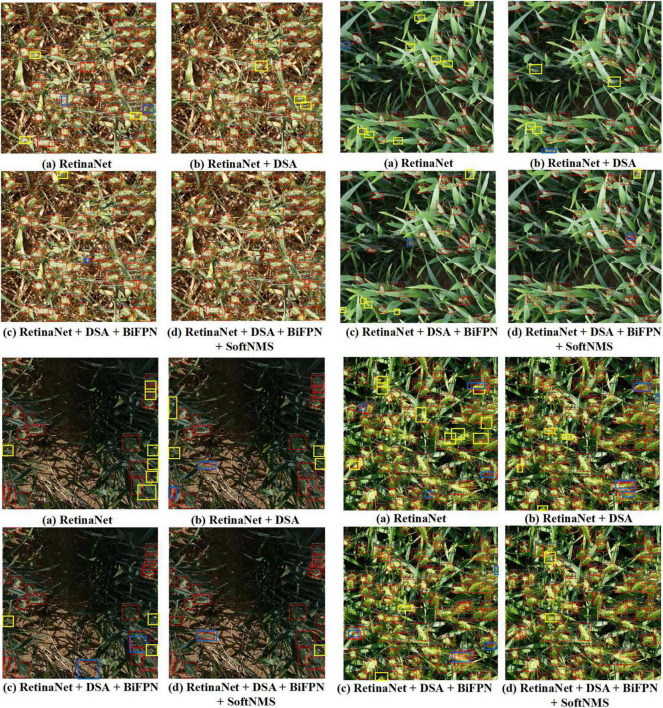
Four different examples, each with the four different methods. **(a)** Results of wheat spike detection with RetinaNet. **(b)** Results of wheat spike detection with RetinaNet + DSA. **(c)** Results of wheat spike detection with RetinaNet + DSA + BiFFPN. **(d)** Results of wheat spike detection with our method. Additionally, yellow boxes are missed spikes, blue boxes are false spikes.

After detection, 60 images of three categories (low illumination, complex environment background, and overlapping occlusion) are selected for counting, with a total of 1,448 wheat spikes. The counting result of our method is 1,345, and the counting accuracy is 92.88%. The counting result of RetinaNet is 1,301, and the counting accuracy is 89.84%. So, our method has improved by 3.04%. The above experiments indicated that our method could effectively overcome the three kinds of difficult recognition images to improve the precision of spike detection. As can be seen from the following four images (the above two images show a complex environment background, and the next two are low illumination and overlapping occlusion), the counting results of four different networks in the same image are inconsistent (Figure 7). Among them, yellow is missing spikes and blue is false spikes. The real counting result is 211, the total counting result of the RetinaNet is 193, and the RetinaNet-DSA-BiFPN result is 205. RetinaNet-DSA-BiFPN [Figure 7(c)] can detect wheat spikes that cannot be detected in RetinaNet [Figure 7(a)], which indicates that the increased fusion channel makes the fusion information more useful. Finally, the total number of our method [Figure 7(d)] is 207. The four images show that the missed boxes of our method are lower than those of other models. This is because Soft-NMS reduces the score of boxes with high IoU rather than directly filtering them out, thus allowing the correct boxes to be retained. The results show that our method improves detection accuracy by 6.63% in images with high detection difficulty.

**FIGURE 8:**
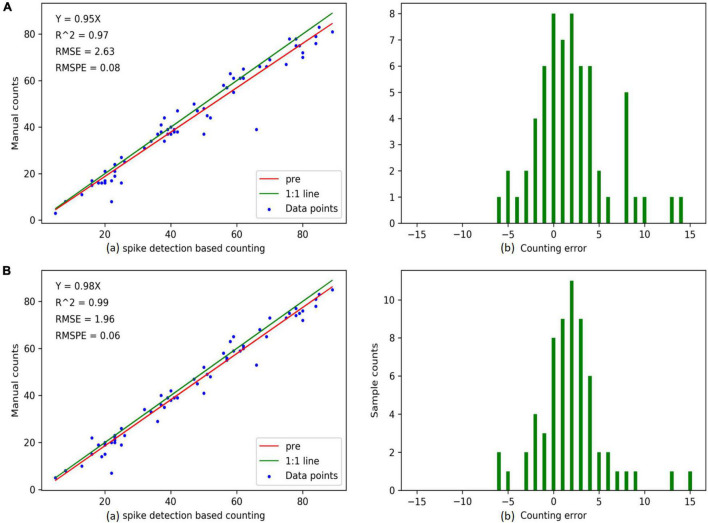
Counting accuracies calculated using “spike detection based counting” **(top row)** and “manual counting” **(bottom row)** strategies, respectively, for individual spikes (a total of 60 data points). The above two images are the results of RetinaNet, and the following two images are the results of our method. **(A(a),B(a))** are linear regression results between the imaging derived and manual counts. **(A(b),B(b))** are the histograms of counting errors. The “pre” in graph “spike detection-based counting” indicates the predicted regression line.

For the detection results of 60 images, a comparison between the “RetinaNet” and the “our method” is performed (Figure 8). The regression slope of “our method” is higher than that of “RetinaNet.” In addition, it has a higher correlation, lower RMSE and RMSPE (the RMSE and the RMSPE of our method are 1.96 and 0.06, the RMSE and the RMSPE of RetinaNet are 2.63 and 0.08), which indicates that the counting result of our method (Figure 8B) is better than that of RetinaNet (Figure 8A). At the same time, in our method, the counting error is concentrated between ±5, which is better than RetinaNet.

### Comparing Against the State-of-the-Art Detectors

**FIGURE 9:**
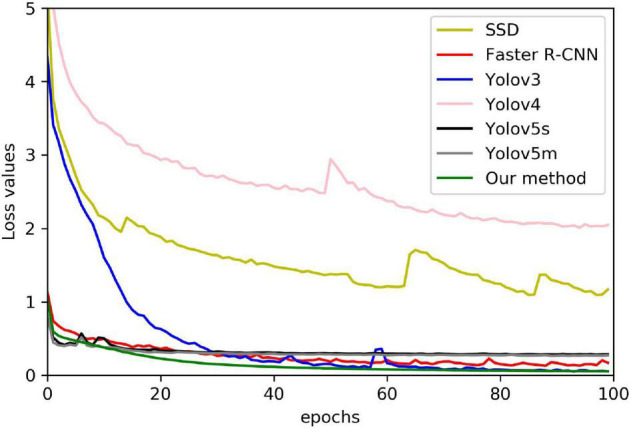
Our method comparing against the state-of-the-art loss.

With mainstream object detection, the one-stage detector used Yolov3, Yolov4, Yolov5s, Yolov5m, and SSD, and the two-stage detector used Faster-RCNN. RetinaNet model is different from the five improved ideas but also has a good detection effect. Figure 9 shows 100 epoch performances of all models, our method, Yolov3, Yolov4, Yolov5s, Yolov5m, SSD, and Faster-RCNN. The convergence speed of the loss value of our method is faster than Yolov3, Yolov4, Yolov5s, Yolov5m, SSD, and Faster-RCNN. The final loss of our method is 0.05, Yolov3 is 0.07, SSD is 1.51, Faster-RCNN is 0.15, Yolov4 is 2.04, Yolov5s is 0.28, and Yolov5m is 0.27. Because the one-stage detector does not deal with the detection frame, the initial value of Yolov3 and SSD loss function is greater. As a result of the imbalance between positive and negative examples, the initial value and overall trend pair differ from the other models. Because of the two-stage detector’s special RPN network, the convergence speed of Faster-RCNN is slower than that of RetinaNet. Due to our model improving on the loss function, the convergence speed of our model is comparable to Yolov5 and better than the other models. Finally, the mAP value also shows that our method has achieved good experimental results (Table 3). The mAP value of our method is 2.79% higher than Yolov3, 1.35% higher than Yolov4, comparable to Yolov5, 7.26% higher than Faster-RCNN, and 49.06% higher than SSD.

**TABLE 3:** Mean average precision (mAP), frames per second (FPS), root mean square error (RMSE), and root mean square percentage error (RMSPE) of SSD, Yolov3, Yolov4, Yolov5s, Yolov5m, Faster R-CNN, and our method in detecting wheat spikes.

Method	Backbone	Datasets	mAP50	mAP75	FPS	RMSE	RMSPE	Counting Acc
SSD	VGG	Mixed	0.4356	0.1652	60	10.30	0.26	0.4841
Yolov3	DarkNet53 + FPN	Mixed	0.8983	0.4832	50	2.56	0.08	0.8991
Yolov4	CSPDarkNet53 + PANet	Mixed	0.9127	0.4902	52	2.13	0.14	0.9095
Yolov5s	CSPDarkNet53 + PANet	Mixed	0.9272	0.5128	60	1.71	0.12	0.9302
Yolov5m	CSPDarkNet53 + PANet	Mixed	0.9312	0.5217	50	1.53	0.06	0.9330
Faster-RCNN	ResNet101 + FPN	Mixed	0.8536	0.4956	10	3.14	0.07	0.8805
**Our method**	ResNet101 + BiFPN	**Mixed**	**0.9262**	**0.5023**	**22**	**1.96**	**0.06**	**0.9288**

*The results of our method are highlighted in bold.*

**FIGURE 10:**
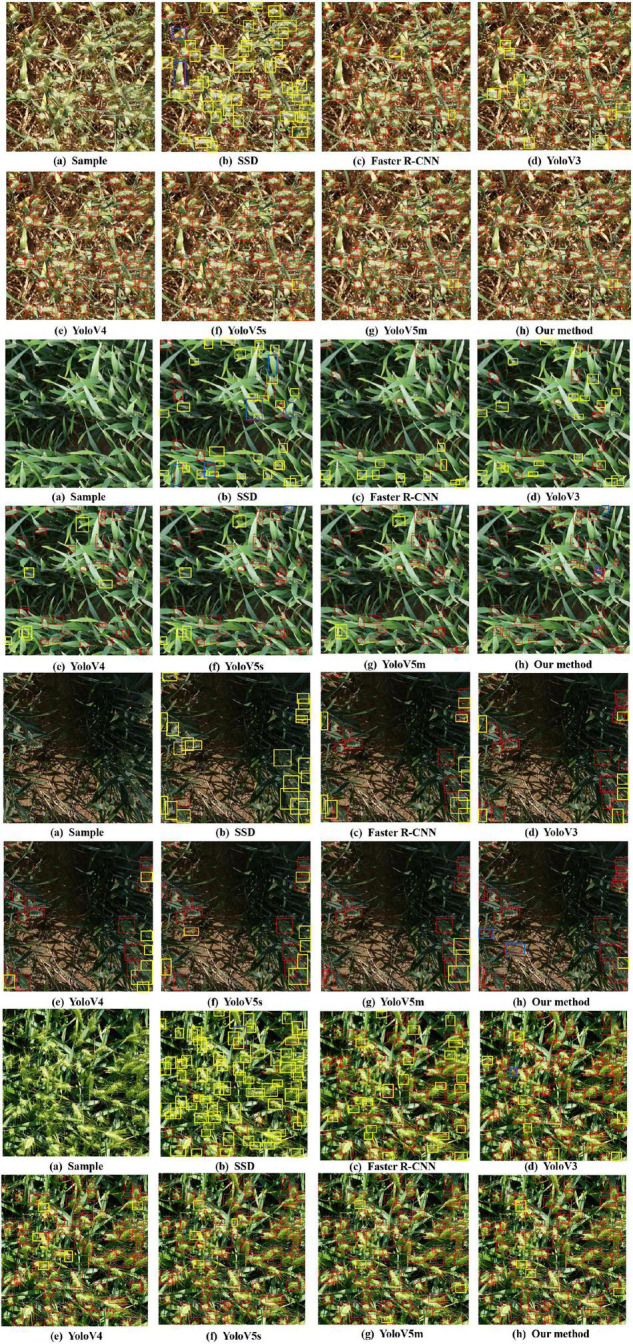
Four different examples, each with the seven different methods: **(a)** sample of images; **(b)** results of wheat spike detection with SSD; **(c)** results of wheat spike detection with Faster R-CNN; **(d)** results of wheat spike detection with Yolov3; **(e)** results of wheat spike detection with Yolov4; **(f)** results of wheat spike detection with Yolov5s; **(g)** results of wheat spike detection with Yolov5m; **(h)** results of wheat spike detection with our method. Additionally, yellow boxes are missed spikes, and blue boxes are false spikes.

After detection, 60 images of three categories (low illumination, complex environment background, and overlapping occlusion) are selected for counting, with a total of 1,448 wheat spikes. The counting result of the Faster-RCNN is 1275, SSD is 701, Yolov3 is 1302, Yolov4 is 1317, Yolov5s is 1347, Yolov5m is 1351, and the counting result of our method is 1345. The counting accuracy of our method is 92.88%, Faster R-CNN is 88.05%, Yolov3 is 89.91%, Yolov4 is 90.96%, Yolov5s is 93.02%, Yolov5m is 93.30%, and SSD is 48.41%. The counting accuracy of our method is 4.83% higher than that of Faster-RCNN, 2.97% higher than that of Yolov3, 1.92% higher than that of Yolov4, and 44.47% higher than that of SSD, and comparable to YoLov5. The above experiments show that our method can effectively overcome the three kinds of difficult recognition images to improve the accuracy of spike detection. As can be seen from the following four images (the above two images show a complex environment background, and the next two are low illumination and overlapping occlusion), the counting results of seven different networks in the same image are inconsistent (Figure 10). Among them, yellow is missed spikes, and blue is false spikes. The real counting result is 211, our method counting result is 207, the Yolov3 result is 170, Yolov4 result is 191, Yolov5s result is 194, Yolov5m result is 200, Faster R-CNN result is 161, and the SSD result is 46. The detection results indicate that Faster-RCNN is not good for images with complex environment backgrounds, Yolov3 and Yolov4 are not good for images with similar background color and occlusion spikes, and the counting effect of SSD is very bad. Additionally, our method is most concentrated in the counting error, mainly between −5 and 10. Therefore, our method is superior to the four methods and comparable to YoLov5.

**FIGURE 11:**
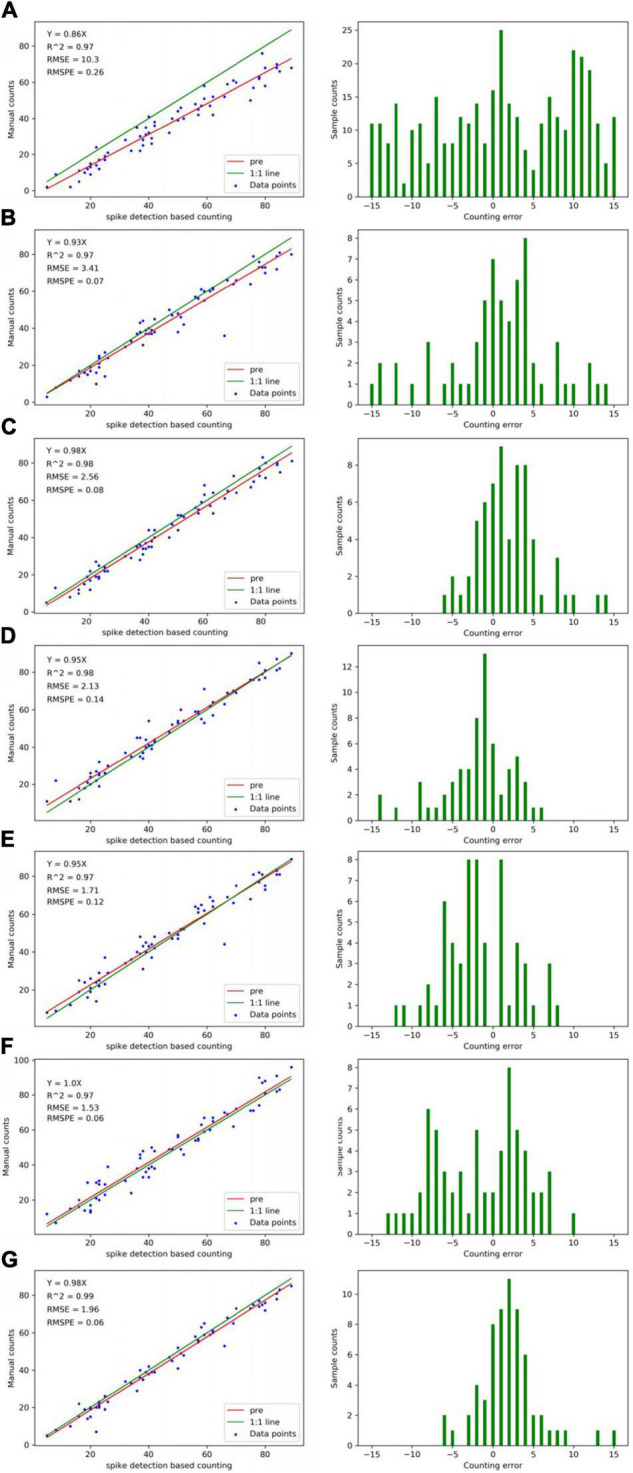
Counting accuracies calculated using “spike detection based counting” (top row) and “manual counting” (bottom row) strategies, respectively, for individual spikes (a total of 60 data points). The **(A)** is SSD, **(B)** is Faster R-CNN, **(C)** is Yolov3, **(D)** is Yolov4, **(E)** is Yolov5s, **(F)** is Yolov5m, and **(G)** is our method. The left is linear regression results between the imaging derived and manual count. The right is the histograms of counting error. The “pre” in graph “spike detection-based counting” indicates the predicted regression line.

For the detection results of 60 images, the comparison among “Faster-RCNN,” “Yolov3,” “Yolov4,” “Yolov5s,” “Yolov5m,” “SSD,” and “our method” is performed (Figure 11). The RMSE and RMSPE of our method are 1.96 and 0.06. Faster R-CNN is 3.14 and 0.07, Yolov3 is 2.56 and 0.08, Yolov4 is 2.13 and 0.14, Yolov5s is 1.71 and 0.12, Yolov5m is 1.53 and 0.06, and SSD is 10.3 and 0.26. The results indicate that our method has better detection and counting effect than Faster R-CNN, Yolov3, Yolov4, and SSD in the mixed dataset.

## Conclusion

In this article, we developed a wheat spike detection method based on the SpikeRetinaNet to address the issue of small dense object detection and counting in complex scenes. The method consists of three critical steps: use BiFPN to better integrate multiscale features, network refinement by adding a DSA block, and Soft-NMS was used to solve the occlusion problem. In addition, the WSD images are added to enrich the varieties of the wheat dataset. Based on the methodology, mAP of wheat spikes and counted were outputted, with detection rates of 92.62 and 92.88%, respectively. Therefore, the knowledge generated by this study will greatly aid in the detection and counting of wheat spikes in complex field environments and provide technical reference for agricultural wheat phenotype monitoring and yield prediction.

## Data Availability Statement

The original contributions presented in the study are included in the article/Supplementary Material, further inquiries can be directed to the corresponding author.

## Author Contributions

CW and CY performed conceptualization and supervised the manuscript. CW and JW carried out methodology, provided the software, validated the manuscript, carried out formal analysis, participated in writing, reviewing, and editing, and contributed in visualization. HS and XC investigated the study. ZL and JW provided the resources. CY and JW contributed in data curation. CW and HC involved in writing original draft preparation. CW involved in project administration and contributed in funding acquisition. All authors have read and agreed to the published version of the manuscript.

## Conflict of Interest

The authors declare that the research was conducted in the absence of any commercial or financial relationships that could be construed as a potential conflict of interest.

## Publisher’s Note

All claims expressed in this article are solely those of the authors and do not necessarily represent those of their affiliated organizations, or those of the publisher, the editors and the reviewers. Any product that may be evaluated in this article, or claim that may be made by its manufacturer, is not guaranteed or endorsed by the publisher.
