# Supplementary figures and images for: Wheat Spike Detection and Counting in the Field Based on SpikeRetinaNet
Source: Front Plant Sci. 2022 Mar 3;13:821717. doi: 10.3389/fpls.2022.821717 (PMC8928106; doi:10.3389/fpls.2022.821717)

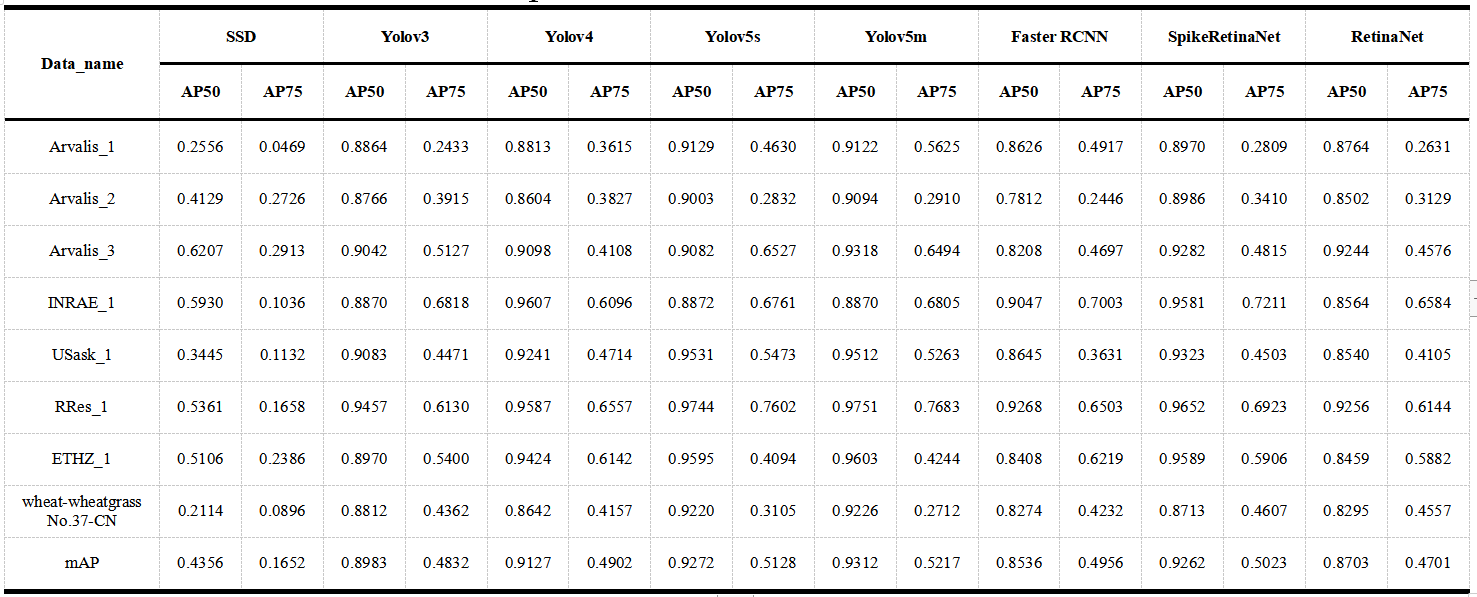

Supplement: Supplementary file 1 [file Image_1.PNG]
